# Investigation on Vortex-Induced Vibration Experiment of a Standing Variable-Tension Deepsea Riser Based on BFBG Sensor Technology

**DOI:** 10.3390/s19153419

**Published:** 2019-08-04

**Authors:** Peng Li, Aijun Cong, Zhengkai Dong, Yu Wang, Yu Liu, Haiyan Guo, Xiaomin Li, Qiang Fu

**Affiliations:** 1 College of Architecture and Civil Engineering, Shandong University of Science and Technology, Qingdao 266590, China; 2 College of Engineering, Ocean University of China, Qingdao 266100, China; 3 Yantai CIMC Raffles Ocean Engineering Co., Ltd., Yantai 264000, China

**Keywords:** deepsea riser, vortex-induced vibration, top tension, BFBG sensor technology, experimental test

## Abstract

A vortex-induced vibration (VIV) experiment on a standing variable-tension deepsea riser was conducted to investigate the applicability and sensitivity of Bare Fiber Bragg Grating (BFBG) sensor technology for testing deepsea riser vibrations. The dominant frequencies, dimensionless displacements, in-line and cross-flow couplings of the riser VIV under different top tensions were observed through wavelet transform and modal decomposition. The result indicated that, excited by the same external flow velocities, the cross-flow and in-line dominant frequencies of the riser both decreased with increasing top tension. In terms of displacement responses, increasing top tension caused the root mean square (RMS) displacement to decrease and the vibration amplitude to reduce. In terms of cross-flow and in-line coupling, the closer a location is to the ends of the riser, the smaller the trajectory is and the more standard the “8” becomes. During top tension increases, there exists a “lag” in the time when the riser’s vibration trajectory becomes an “8”. The Slalom Surround Installation approach can effectively prevent the local breakage of the optical fiber string. BFBG sensor technology can give an accurate presentation of the displacement time history, vibration amplitude and frequency of the riser, provides a clear picture of how the riser’s mode and VIV evolve as a function of flow velocity.

## 1. Introduction

Top-tensioned risers are a unique form of riser application that tensions the riser by applying tension through a top tensioner. They usually serve as a component for linking dynamic floating units to the underwater systems. When ocean currents flow past these slender, cylindrical structures, at a given flow velocity, alternately discharged vortexes at the tail will form cyclic wake. Owing to the cyclicity and asymmetry of vortex shedding, the fluid will exert a cyclic pulsating force to the structure in both in-line and cross-flow directions, causing it to vibrate in both directions. This is what we call vortex-induced vibrations (VIV) [1,2]. When the natural frequency of the riser structure is close to the vortex shedding frequency, vibration will force the vortex shedding frequency to lock in somewhere near the natural frequency of the structure, referred to as a ”lock-in”, thereby intensifying the cross-flow vibration of the riser. This “lock-in” will accelerate the fatigue damage of the riser.

In order to reduce or eliminate the damaging effect caused by the change of vortex-induced vibration caused by the change of top tension and prolong the service life of deepsea riser, scholars from various countries have conducted extensive research. Chaplin et al. measured the VIV of a top-tensioned riser undergoing stepped currents in a water flume [3]. Lie and Kaasen investigated the VIV of a top-tensioned riser at a port by measuring the modal response of the riser and the in-line and cross-flow VIV of the riser under large aspect ratios [4]. Huera-Huarte and Bearman looked at the wake flow structure and VIV responses of a flexible cylinder and examined how top tension affects the phase-locked vibration response bifurcation of the riser model [5,6]. Lee and Allen tested the VIV of a top-tensioned flexible cylinder and demonstrated that top tension and structural rigidity can play a critical role in vibration frequency [7]. Srinil et al. investigated the impacts of riser’s fluid-solid coupling parameters on riser’s nonlinear dynamics under different sheared currents through numerical VIV simulation and prediction of variable-tension vertical flexible risers in linearly sheared currents [8]. The researchers used different theories and analytical methods to test the dynamic response of the riser under different top tensions. Among them, Gao et al. analyzed the VIV response and parameters of full-size, tension deepsea risers under non-locked-in conditions with a model based on the Vander Pol theory. They concluded that the riser’s inherent frequency in in-line direction is higher than that in cross-flow direction and top tension variation makes a great difference to the riser’s inherent frequency and vibration mode [9]. Li et al. was the first to apply vector finite element method to the dynamic behavior analysis of top tension risers [10]. They derived the motion formulation for riser particles by simulating the inter-particle interaction with plane bending bar elements. They developed a Matlab solver, calculated the dynamic response characteristics of the riser under ocean currents and waves and compared the result with measurements from traditional finite element method. Zhang et al. converted the partial differential equation of motion for deepsea tension risers into a Mathieu equation using the Gallerkin procedure [11]. They yielded the instability diagram of the riser by stacking each separate order of mode of the riser against this Mathieu instability diagram and compared the result with the numerical computation result based on the Floquet theory. Zhang et al. used the discrete control equation of finite element method and solved it in the time domain using the Newmark-β approach [12]. They examined the limit displacement, bending moment, stress, and top and bottom corners of a top-tensioned riser subjected to up-convex internal isolated wave. They analyzed the impacts of the amplitudes of the internal isolated wave, density difference between adjacent layers, top tension, and wall thickness on riser response. 

Fiber Bragg grating (FBG) sensors are a new sensor technology that is superior over its traditional counterparts due to small volume, light mass, simple head construction, high capacity, good stability, simple wire routing, corrosion rigidity, high precision, resistance to electromagnetic interference (EMI), and flexibility for remote transmission and distributed measurement [13,14,15]. As it avoids the exposure to EMI and the null drift potential in small structural strain cases for electrometric techniques, this new technology is more applicable for marine projects subjected to humidity, corrosion, and complex EMI. As FBG sensors are small in volume and light in mass, their extra mass to the riser model is ignorable. Besides, they are also suitable for underwater operations. As a number of measuring points have to be deployed along the length of a deepsea riser to enable VIV modal analysis, the greatest advantage of using FBG sensors is that a number of gratings can be written into one single fiber to make up a sensor array and allow for distributed sensing. This eliminates the need of large amounts of lead wires, the difficulty of routing the wires, and the need of numerous data acquisition and analysis interfaces challenging traditional electrometric sensors. Moreover, excessive lead wires would cause the fluid around the riser to produce severe disturbance, which could then modify the initial external flow field and greatly limit the data accuracy. When traditional FBG sensors are used, as fibers have very low bending strengths, the wires are usually encapsulated. As the cross section of the fiber is larger after it is encapsulated, when installed on the surface of a large aspect ratio riser, it will affect the flow state surrounding the riser and change the dynamic response characteristics of the riser itself. Therefore, the use of Bare Fiber Bragg Grating sensor technology is an ideal choice for deep sea riser VIV testing, but a series of core technologies such as the sensitive testing, positioning, installation of Bare Fiber Bragg Grating sensors, the improvement of fiber flexural strength and the development of new coating material shave not yet been broken, which has great constraints on the long-term stability of FBG sensors in the marine environment.

As reviewed above, we consider the limitations of the existing research mainly focused on the limitation of the top tension as a factor limiting the rotating motion of the riser. Therefore, in this paper, we explore the applicability and sensitivity of Bare Fiber Bragg Grating (BFBG) sensing technology in vibration testing of long and thin marine structures such as deepsea risers. In this study, we carried out a VIV experimental test on a standing variable-tension riser subjected to a uniform flow in the combined wave–current water flume of the Engineering Hydrodynamics Laboratory, Ocean University of China. In this study, we used BFBG sensors to observe the VIV dominant frequency, dimensionless displacement, and in-line and cross-flow coupling of the variable-tension riser by varying the external flow velocity and top tension and using wavelet transform (WT) and modal decomposition.

## 2. Bare Fiber Grating Sensing Technology and Data Analysis Method

### 2.1. Working Principle of BFBG Sensor Technology

**Figure 1:**
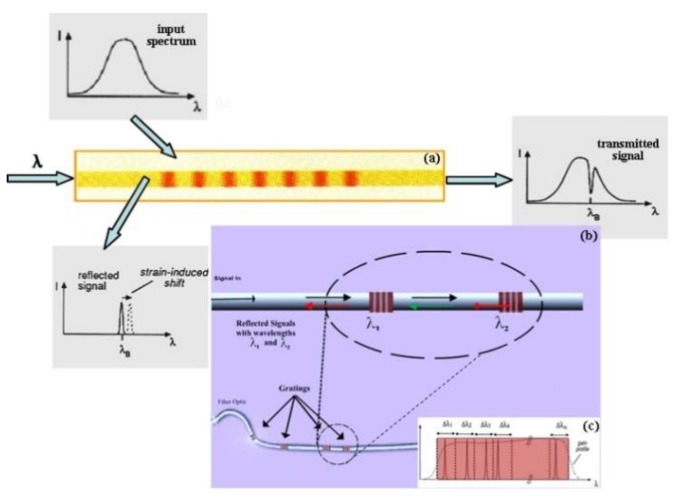
Schematic diagram of the operation and Select configuration diagram of the FBG sensor. (**a**) Working principle of Fiber Bragg grating (FBG); (**b**) wavelength division multiplexing (WDM) technology for FBG sensors; (**c**) Selection and configuration of FBG sensors.

The Fiber Bragg Grating (FBG) sensor is a wavelength modulation type optical fiber sensor that obtains sensing information by modulating the optical fiber Bragg wavelength by external physical parameters, and is made by utilizing the photosensitivity of the optical fiber material. In principle, it belongs to a light reflecting type device and a fiber grating sensor. The working principle is shown in Figure 1 [16].

The Fiber Bragg Grating (FBG) sensor is made of the photosensitivity of optical fiber material. The continuous broadband light emitted by the light source through the optical fiber is coupled with the optical field, so that the broadband light is selectively reflected back to a corresponding narrow band light and returned along the original transmission fiber. The remaining broadband light is transmitted directly through the past.

When the temperature or strain used for the fiber grating changes, the wavelength of the narrow-band light center reflected back will change linearly. According to the coupled mode theory, only the light wave that satisfies the Bragg condition can be reflected, which is expressed as:

*λ_B_ = 2n_eff_* Λ. (1)

where *λ_B_* [17] is the center wavelength of the reflected light wave of the FBG, *n_eff_* is the effective refractive index of the core, and Λ is the fiber grating period.

When the temperature and stress of the fiber grating change, the core effective refractive index *n_eff_* or the grating period Λ changes, and the central reflection wavelength also changes accordingly, which is expressed as:

(2) $$\Delta \lambda_{B}=(1-P_{e})\Delta \varepsilon \lambda_{B}+(\alpha_{f}+\xi )\Delta T\lambda_{B}$$

where Δ*λ_B_* is the amount of change in the center wavelength of the reflected light wave [18], *Pe* is the effective elastic coefficient of the fiber, Δε is the amount of strain change, ΔT is the amount of temperature change, *α_f_* is the thermal expansion coefficient of the fiber, and ξ is the thermo-optic coefficient of the fiber.

Equation (2) reflects that the amount of change in the center wavelength of the fiber grating is linearly related to the strain change amount or the temperature change amount, and the corresponding strain or temperature variable can be obtained by inverse calculation by the above formula. It can be known from the formulas (1) and (2) that the effective elastic coefficient *Pe* is an important action parameter for the strain caused by the stress change, and can be described as the amount of refractive index change due to stress or strain. Based on the principle and composition of the FBG sensor, it has the characteristics of high sensitivity, corrosion resistance, high and low temperature resistance and electromagnetic interference resistance. For the typical long-structure structural fluid-solid coupling test of deepsea riser VIV test, the above characteristics are especially important.

### 2.2. Data Analysis Methods

#### 2.2.1. Analysis of Strain Data

In the process of strain data acquisition in the oscillating interval, in order to eliminate the initial strain caused by the top tension and the axial strain during the vibration of the riser, the bending strain of the riser caused by VIV is accurately collected, and the BFBG strain sensors are separately arranged. Four sets of bare fiber strings were used to engrave grating measuring points along the length of the fiber string according to the test scheme, and the dynamic strains in the cross-flow and in-line directions were measured. The strains obtained from the two measuring points in the symmetrical position are averaged to obtain the bending strain of the riser, as shown in Equations (3) and (4):

(3) $$\varepsilon_{CF}\left(t\right)=\left[\varepsilon_{CF-2}\left(t\right)-\varepsilon_{CF-4}\left(t\right)\right]/2$$

(4) $$\varepsilon_{IL}\left(t\right)=\left[\varepsilon_{IL-1}\left(t\right)-\varepsilon_{IL-3}\left(t\right)\right]/2$$

where: $\varepsilon_{CF}\left(t\right)$, $\varepsilon_{IL}\left(t\right)$ is the cross-flow and in-line direction bending strain of the riser caused by vortex-induced vibration; $\varepsilon_{CF-2}\left(t\right)$, $\varepsilon_{CF-4}\left(t\right)$ corresponds to two cross-flow directions. The strain time history obtained by the sensor; $\varepsilon_{IL-1}\left(t\right)$, $\varepsilon_{IL-3}\left(t\right)$ is the strain time history obtained by eliminating the initial bending strain corresponding to the two sensors in the in-line direction.

#### 2.2.2. Time Frequency Analysis Method

Wavelet transform (WT) is a new transform analysis method that overcomes the shortcomings of window size and frequency variation. It provides a “time-frequency” window with frequency change, which is ideal for signal time-frequency analysis and processing tool. The continuous WT equation is shown as follows:

(5) $$W(a,\tau )=a^{-1/2}\int_{-\infty }^{\infty }f(t)\Psi^{*}(\frac{t-\tau }{a})dt$$

where, W(a,τ) means the coefficient obtained after the function f(t) is subject to wavelet transform or the value of change in frequency in terms of time scale; *a* means scale factor; *τ* means shift factor; Ψ(t) means mother wavelet function. The mother function used is Morlet complex wavelet, which is defined as follows:

(6) $$\Psi (t)=e^{i\omega t}e^{-t^{2}/2}$$

Its Fourier transform is expressed as follows:

(7) $$\Phi (\omega )=\sqrt{2\pi }e^{-1/2{\left(\omega -\omega_{0}\right)}^{2}}$$

The coefficient matrix W(a,τ) obtained after WT was obtained. The actual frequency function f(t) is returned to according to transform scale. The change in the color depth shown on the time frequency plots is the vibration intensity under the frequency. According to our analysis, the change in the intensity of frequency within time domain and the laws of changes in the characteristic frequency of VIV can be reflected on the time frequency plots. Also, frequency components and the instability of VIV in time sequence can be presented on the plots.

#### 2.2.3. Strain Signal Based Modal Analysis Theory

For a riser with the length of *L* that is articulated at both ends, the vibration displacement of the structure at any time *t* can be expressed as y=y(z,t) According to the modal decomposition of structural dynamics theory [19], the displacement at the time *t* can be expressed by the following equation:

(8) $$y(z,t)=\sum_{n=1}^{\infty }\omega_{n}(t)\varphi_{n}(z)\;z\in [0,L].$$

where, $\varphi_{n}(z)$ means vibration mode function; and $\omega_{n}(t)$ means weight coefficient.

For a riser structure that is articulated at both ends, its vibration mode function $\varphi_{n}(z)$ can be expressed as the following sine function:

(9) $$\varphi_{n}(z)=\sin\frac{n\pi z}{L}\;z\in [0,L].$$

Thus, the displacement function can be expressed as follows:

(10) $$y(z,t)=\sum_{n=1}^{N}\omega_{n}(t)\sin\frac{n\pi z}{L}\;z\in [0,L].$$

By solving the weight function corresponding to each mode $\omega_{n}(t)$, the displacement time history of each point of the riser can be calculated.

## 3. Experimental Set-up

### 3.1. Experimental Apparatus

**Figure 2:**
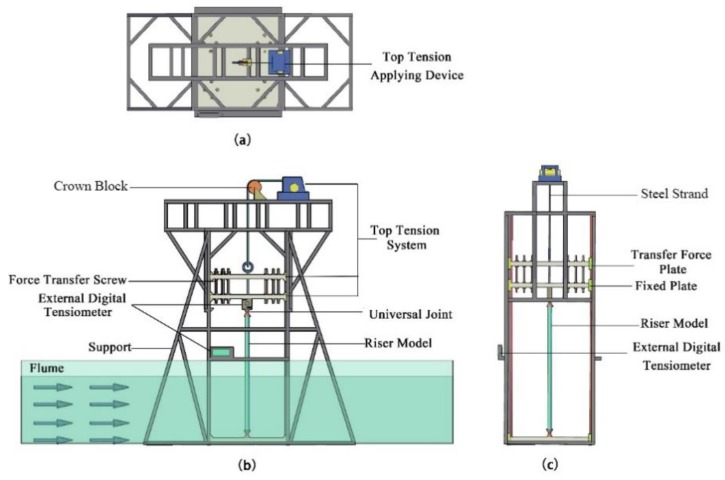
General setup of the experiment (**a**) Top view; (**b**) Elevation view; (**c**) Side view.

The experiment was carried out in the combined wave–current water flume of the Engineering Hydrodynamics Laboratory, Ocean University of China. The flume has a cross section of 1.0 × 1.2 m, a maximum working water depth of 1 m and a maximum external flow velocity of 1 m/s. The working water depth is 0.8 m, and the flow velocity is 0.1–0.6 m/s. The flume is sided with toughened glass, partitioned with steel columns and bottomed with steel plate which can be used for fixing the riser support. The entire system can be used to simulate regular flow velocities and precisely vary the top tension. Figure 2 shows the general setup of the experiment.

**Figure 3:**
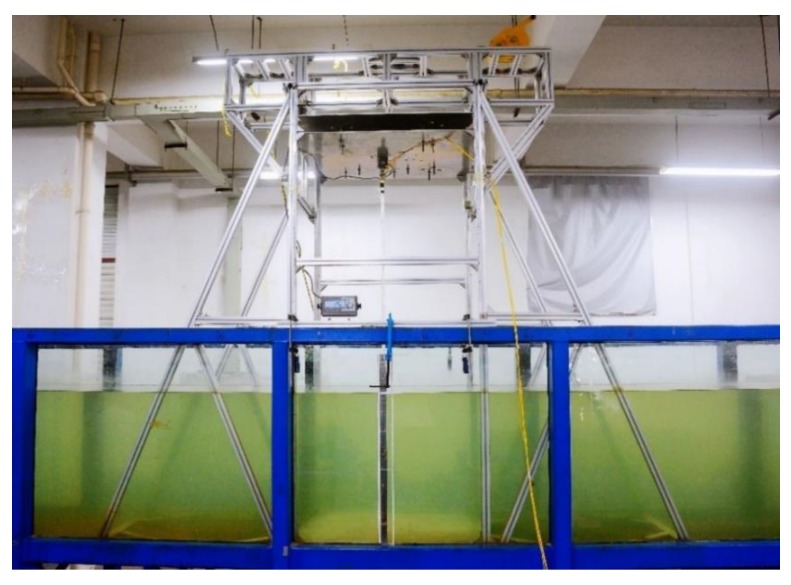
Actual working conditions of installation and launching of riser and supporting structure.

**Figure 4:**
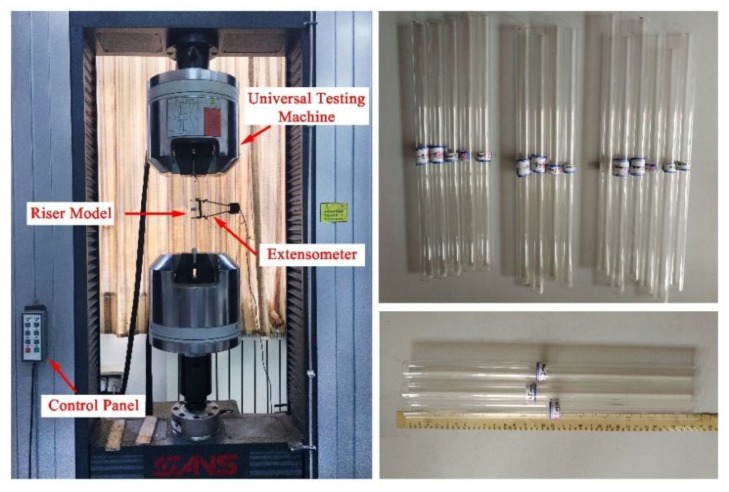
Experimental model and mechanical properties test.

The test apparatus consists of a supporting structure, a top tension system, a riser model, and a signal measuring and acquisition system. To ensure accurate application of top tension, a top tension applying system compatible with the supporting structure was designed. Along the upper end of the riser, the top tension system comprises a universal joint, a tensiometer, force transfer screw, steel strand, and a self-locking tensioner. To increase the rigidity of the fixing structure at the upper part of the riser, two equally sized aluminum alloy plates with the same boring positions are fabricated and connected to each other with high-strength power screws along the edge of the plates. The force transfer screw at the upper part of the riser connecting the steel strand is run through the hole in the middle of the two aluminum alloy plates such that tension can be applied to the riser by adjusting the tensioner. The periphery of the aluminum alloy plate is connected to the linear guide via a sliding block such that the plate will slide freely during tension application. When the reading on the tensiometer arrives at the design value, the tensioner will lock itself and lock the force transfer screw connecting the riser in on the aluminum alloy plate; the aluminum alloy plate will be locked in on the linear guide by the ”lock-in” function of the sliding block. This device is designed to prevent unstable load at the end of the riser during external flow velocity variation and effectively control the application of top tension on the riser. Figure 3 shows the actual experimental conditions. The top tension applying system is shown in Figure 4.

### 3.2. Riser Model

The experimental model consists of an organic glass pipe with outside diameter 18.0 mm, wall thickness 1.0 mm, and effective length 2.0 m. Risers were specifically fabricated by a professional manufacturer according to design, separately sampled the selected riser models, and tested the mechanical properties in the universal testing machine. The left picture in Figure 4 shows the pull test of the universal test machine, and the right picture shows the sample of the riser model selected for the test. During the experiment, the riser model was placed in standing posture in a uniform flow with water depth 0.8 m. Table 1 details the parameters used for the experiment.

### 3.3. BFBG Sensors

**Figure 5:**
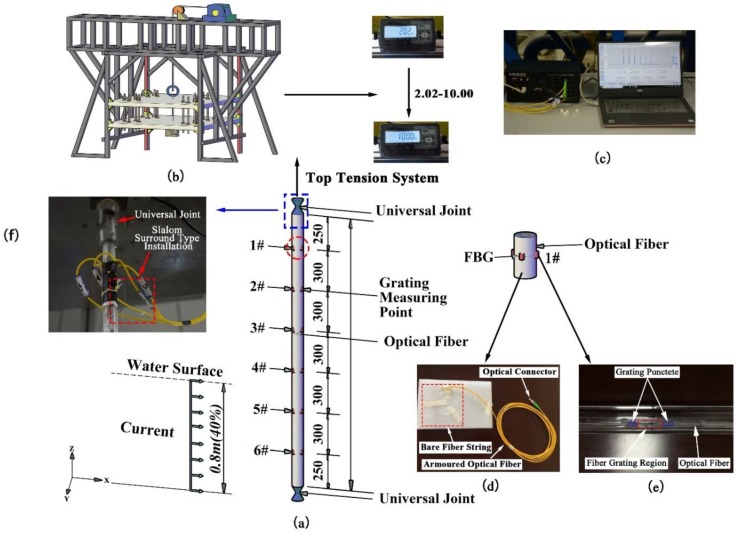
Layout of the top tension system and FBG sensors. (**a**) Riser model; (**b**) Control diagram of the top tension system; (**c**) FBG demodulator; (**d**) Bare fiber string and armored optical fiber; (**e**) Layout of FBG measuring points; (**f**) Installation of bare fibers.

A high-definition digital camera was used to monitor riser vibration and to take photos at the end of the sampling time. Top tension was varied by adjusting the tension applying device, as illustrated by Figure 5b. Bare Fiber Bragg Grating (BFBG) sensors were used for the experiment. Four strings of bare fibers were deployed symmetrically along the surface of the riser. Six grating measuring points were engraved on each fiber string and four FBG sensors were mounted at each point at 90° intervals. A total of 24 measuring points was deployed along the length of the riser model to measure the cross-flow and in-line riser vibration, as illustrated by Figure 5a. As bare fibers have very small diameters––as small as 0.25 mm, high brittleness and limited bending strength, while their serial nature can prevent the potential impact of wire routing, the fibers are extremely prone to rupture damages. Hence, to prevent the bare fibers from rupture during installation, we developed a Slalom Surround Type Installation approach, as illustrated by Figure 5f. This method greatly reduces the rupture potential of bare fibers and well guarantees the overall operational stability and durability of the fiber system during the experiment. Bare fiber strings and a four-channel SM130 FBG demodulator (Micron Optics, Atlanta, GA, USA) were used, as illustrated by Figure 5c,d.

### 3.4. Riser VIV Test Cases under Different Top Tensions

Based on the BFBG sensor technology, five levels of top tension, namely, 19.8 N, 39.2 N, 58.8 N, 78.4 N, and 98.0 N were designed. To avoid potential impacts of vortex discharge induced by the Doppler velocimeter on the experiment and to measure the flow velocity accurately, the Doppler velocimeter was mounted at 2.0 m upstream of the riser. 10 levels of flow velocity spanning from 0.1 to 0.6 m/s were applied at 0.05–0.1 m/s incremental intervals. Top tension was applied to the riser by a top tension applying system consisting of a top tension applying device, a fixed pulley, wire rope, a load plate, a guide rod, a fixing plate, and a AXT-S-100 external digital tensiometer with sensor sensitivity of 1.5–3.0 mV/V. Table 2 details the design experimental cases.

## 4. Results and Discussion

### 4.1. Response Frequency

**Figure 6:**
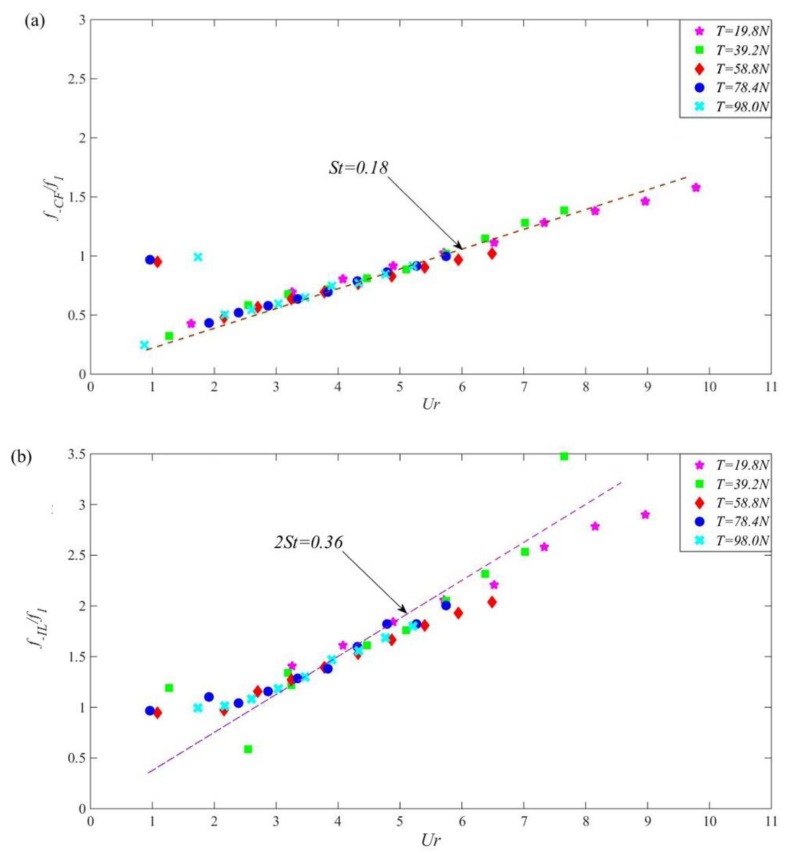
The dimensionless dominant frequency of VIV in the direction of cross-flow (**a**) and in-line (**b**) varies with reduced velocity and Strouhal number fitting under different top tension.

In order to investigate the impacts of top tension on the riser’s dominant frequency, the riser model was fixed in still water. Without changing the other influencing factors, the BFBG strain signals were obtained through external load excitation by varying the external flow velocity at 10 levels; the dominant frequencies of the riser under different cases were yielded through Fourier transform of these strain signals [20]. Figure 6 shows the dimensionless dominant frequencies of the riser in cross-flow (a) and in-line (b) directions versus reduced velocity under different top tensions.

From these diagrams, at *T* = 19.8–98.0 N, when *U_r_* < 3.0, the dimensionless dominant frequency made a “jump” in both directions and is largely deviated from the fitting curve, but the dimensionless dominant frequency in both directions gradually increased with increasing reduced velocity. The cross-flow dimensionless dominant frequencies were linearly fitted using least squares method. The slope ratio was the Strouhal number. Under all five levels of top tension, the Strouhal numbers were not much different but all fluctuated near 0.18; the slope ratios yielded from linear fitting of the in-line dimensionless dominant frequencies all stayed near 0.36. This concurs with the observation that the in-line to cross-flow dominant frequency ratio is approximately 2: 1 [21,22,23].

**Figure 7:**
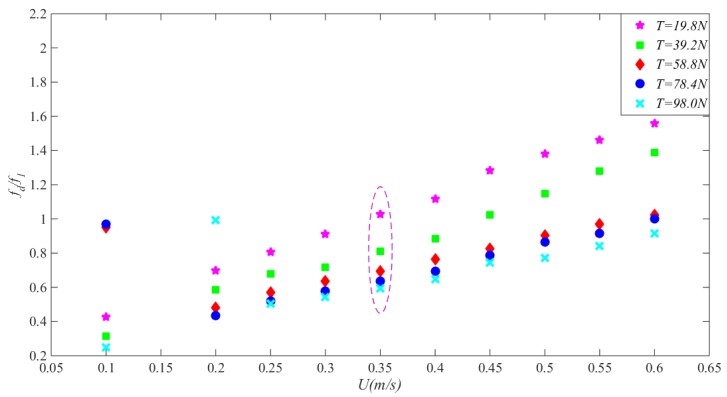
The dimensionless dominant frequency of VIV in the direction of cross-flow varies with the outflow velocity under different top tension.

Figure 7 shows the dimensionless dominant frequency of VIV in the direction of cross-flow varies with the outflow velocity under different top tension. As flow velocity increased, the dominant frequency of the riser gradually increased both in cross-flow and in-line directions under all five levels of top tension. However, under the same flow velocity, as top tension increased level by level, the dominant frequency of the riser gradually decreased, as marked by the purple dotted line in Figure 7.

For the “lock-in” state of riser VIV, it is generally accepted that the theoretical interval is [6.0, 7.5] [24]. As derived, the first-order inherent frequency of the riser under the five levels of top tension was 3.408 Hz, 4.354 Hz, 5.139 Hz, 5.801 Hz, and 6.403 Hz, respectively; consequently, the theoretical external flow velocity interval for ”lock-in” at the corresponding top tensions should be [0.36, 0.46], [0.47, 0.59], [0.56, 0.69], [0.63, 0.78], and [0.69, 0.86], respectively. Hence, four typical levels of external flow velocity, namely 0.35, 0.45, 0.55, and 0.60 m/s, were used to observe how the characteristic frequency of the riser in the ”lock-in” interval evolves under different top tensions. 

**Figure 8:**
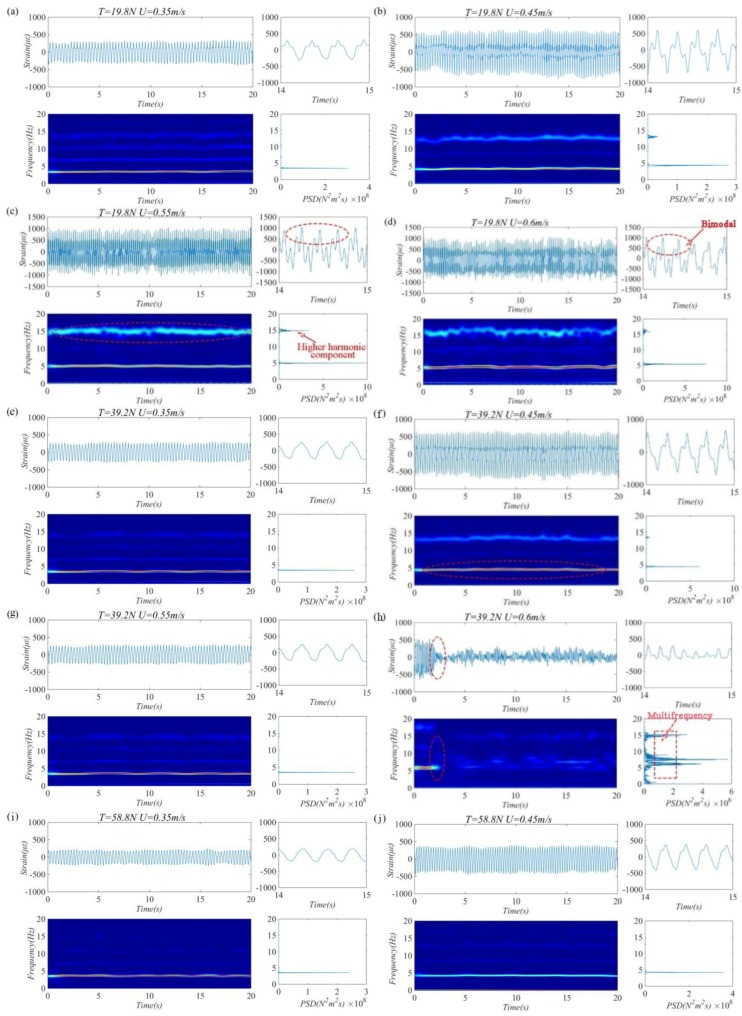

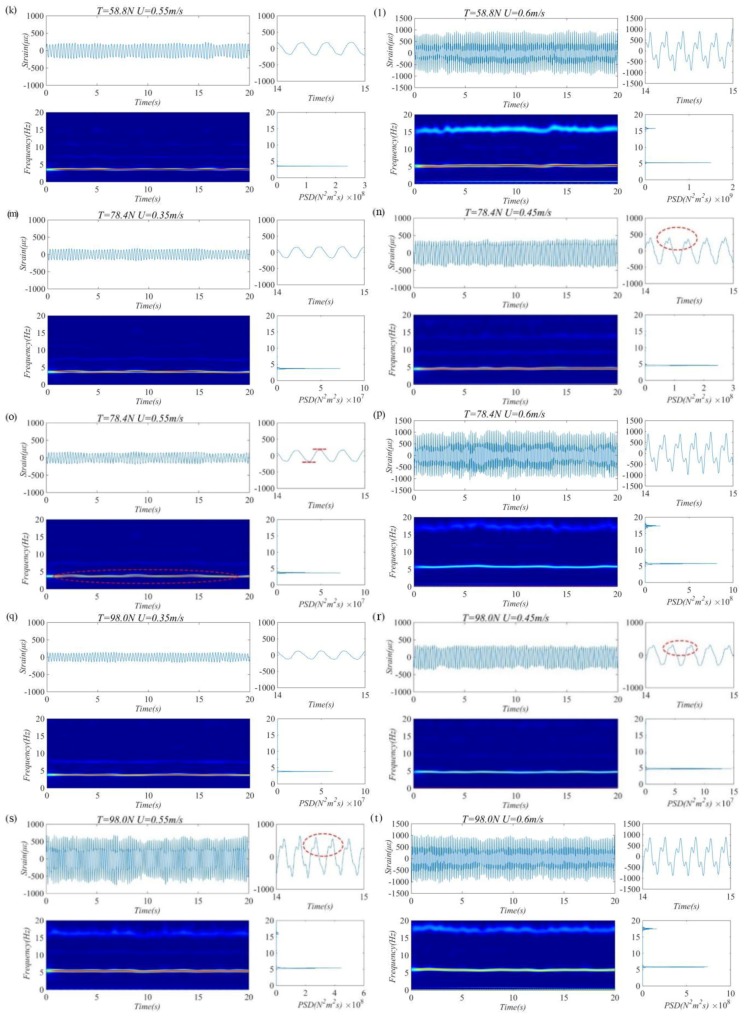
Strain time history (top left), enlargements of strain time history (top right), wavelet time–frequency scale diagrams (bottom left) and power spectral density diagrams (bottom right) of cross-flow riser VIV versus flow velocity under different top tensions (for each level of top tension, (**a**)–(**d**), (**e**)–(**h**), (**i**)–(**l**), (**m**)–(**p**), (**q**)–(**t**), correspond to four flow velocities: 0.35, 0.45, 0.55, and 0.6 m/s).

To give a clear characterization of the transient variation of the riser when entering the ”lock-in” interval, Figure 8 depicts the cross-flow strain time history curve, wavelet time–frequency scale diagrams, and power spectral density curves of the riser VIV versus flow velocity under different top tensions (for each top tension, (a)–(d), (e)–(h), (i)–(l), (m)–(p), (q)–(t), correspond to four levels of flow velocity: 0.35, 0.45, 0.55, and 0.60). From these diagrams, within the tested flow velocity interval, the intensity of third- or higher-order frequency was very small, whereas first- and second-order frequencies had a high involvement; as external flow velocity increased, the intensity of the second-order frequency increased remarkably. This is because the riser had jumped out of the first-order ”lock-in” region and was nearing the second-order ”lock-in”. When *U* > 0.35 m/s, at *T* = 19.8–39.2 N interval, the riser strain displayed a “bimodal” (“Bimodal” means that there are two peaks in the strain time history curve of the riser due to the superposition of strain signals [25].) state in time series, and high-order harmonic components can be observed from the time–frequency diagram. When *T* = 58.8–98.0 N, no obvious “bimodal” was detected from the riser’s strain time history, and the high-order harmonic components in VIV response were no longer remarkable. This suggests that as top tension increased, the cross-flow and in-line coupling of the riser gradually decreased. Interestingly, when *U* = 0.45 m/s, the intensity of first-order vibration was weak at *T* = 19.8 N, 58.8 N, and 98.0 N; at *T* = 39.2 N and 78.4 N, high-vibration red regions appeared across the oscillographic interval; the strain amplitude remained “bimodal”. As top tension increased, the strain amplitude continued to decline and the difference between the two peaks continued to diminish. When *U* = 0.55 m/s, it can be observed that at *T* = 19.8 N, the intensity of the riser’s second-order vibration gradually increased and its distribution was quite concentrated. In this case, the second-order vibration frequency was gradually excited; as top tension increased, the energy region of the second-order vibration frequency was discretized and the intensity decreased. When *U* = 0.6 m/s, almost the same thing happened to the riser’s VIV response as when *U* = 0.55 m/s under all top tensions. In particular, at *T =* 39.2 N, when *U* = 0.6 m/s, the riser’s strain amplitude displayed a sudden local decline. The corresponding vibration frequency was discretized in time series 3–35 s, and a wideband occurred in the power spectral density curve. As analyzed above, after wavelet transform, the time-domain signals obtained by BFBG sensors can accurately describe the dominant frequency and intensity evolutions of risers with different top tensions and gives a true picture of the dynamic responses in and outside the ”lock-in” interval.

### 4.2. VIV Displacement Response Analysis

**Figure 9:**
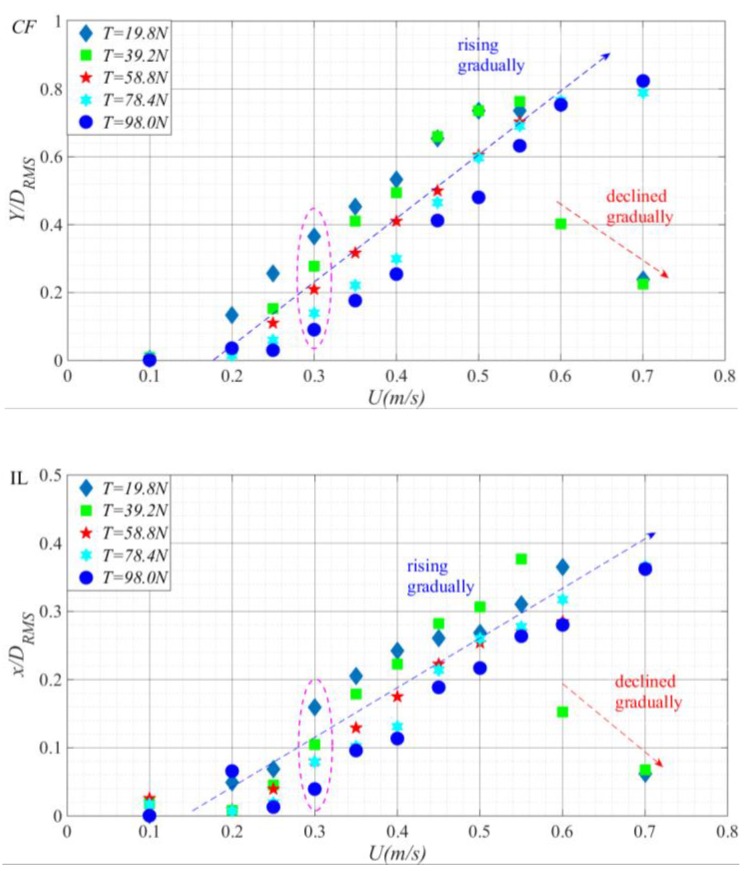
Cross-flow and in-line riser RMS dimensionless displacements versus flow velocity under different top tensions.

Figure 9 compares the in-line and cross-flow RMS dimensionless displacements of the riser versus flow velocity under different top tensions. The purple dotted line delineates the RMS dimensionless displacement curve variation rule of the riser at the same flow velocities under different top tensions. The blue and red imaginary lines represent the RMS dimensionless displacement curves variation rule of the riser versus external flow velocity.

**Figure 10:**
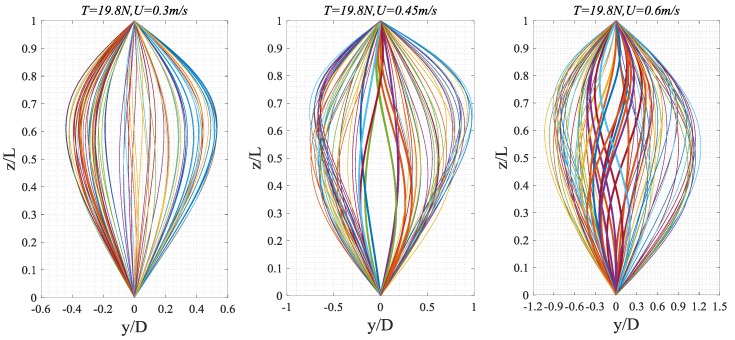
Each (1/20) s of the riser forms a cross-flow deflection curve in motion, *T* = 19.8 N, *U* = 0.3 m/s, *U* = 0.45 m/s, *U* = 0.6 m/s.

From the cross-flow and in-line RMS dimensionless displacements of the riser shown in Figure 9, as top tension increased, the RMS displacement of the riser decreased in both directions. That is, increasing top tension will reduce the riser’s vibration amplitude, as have been reached the corresponding conclusion from Figure 8. At *T* = 19.8–98.0 N, when *U* < 0.55 m/s, as external flow velocity increased, the RMS displacement curve of the riser displayed an increase. In particular, at *T* = 19.8–39.2 N, when *U* > 0.55 m/s, as external flow velocity increased, the RMS displacement curve of the riser displayed a decline. At *T* = 58.8–98.0 N, the vibration increase of the riser was marginally smaller and across the entire external flow velocity interval. This may be attributed to the fact that the increase of the outflow velocity makes the vibration mode of the riser jump from the first order to the second order [26], in Figure 10. When *U* = 0.3 m/s, increasing top tension seemed to cause a gradual longitudinal decline of the RMS dimensionless displacement. In cross-flow direction, when *U* > 0.45 m/s, the riser’s vibration amplitude was quite similar at *T* = 19.8 N and *T* = 39.2 N; when *U* > 0.45 m/s, increasing top tension did not change the vibration of the riser at *T* = 19.8–39.2 N, whereas at *T* = 58.8–98.0 N, as external flow velocity increased, the vibration amplitudes of the riser drew closer; at *U* = 0.6 m/s, the they became the same. This indicates that within this interval, increasing the top tension will increase the RMS displacement growth amplitude of the adjacent two flow velocities in Figure 9.

**Figure 11:**
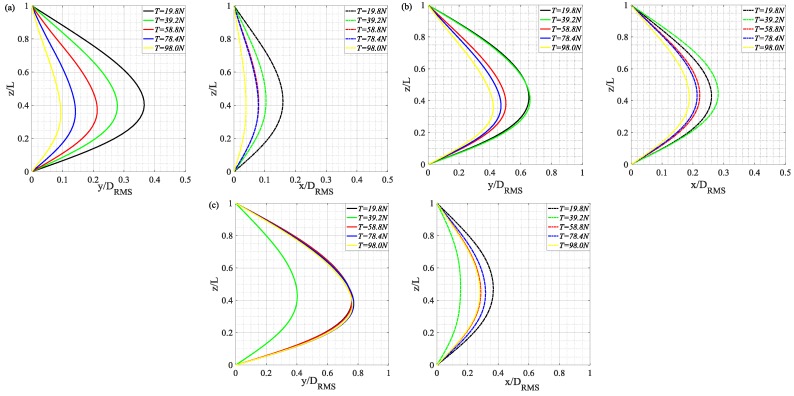
Cross-flow and in-line riser RMS displacements curves along the length of the riser at U = 0.3 m/s (**a**), U = 0.45 m/s (**b**), U = 0.6 m/s (**c**) under different top tensions.

Modal response analysis based on the stable, accurate strain measurements by BFBG sensors yielded the cross-flow and in-line RMS displacements along the length of the riser at *U* = 0.3 m/s (a), *U* = 0.45 m/s (b), and *U* = 0.6 m/s (c) under different top tensions, as shown in Figure 11. Here coordinate (z/L) denotes the normalized riser height. From these graphs, the riser VIV was dominated by first-order mode under all top tensions. When *U* = 0.3 m/s, at T = 19.8–98.0 N, the RMS displacements along the length of the riser in both cross-flow and in-line directions were positively correlated to increasing top tension, which concurs with our previous observations from Figure 9; when *U* = 0.45 m/s, at T = 19.8–98.0 N, increasing top tension no longer made a great difference to the riser’s vibration amplitude; when *U* = 0.6 m/s, in cross-flow direction, the effect of top tension variation on the riser’s RMS dimensionless displacement further degraded and the RMS displacements under T = 19.8 N, 58.8N, 78.4 N, 98.0 N were quite the same. The same happened in in-line direction, too. The results show that the vibration effect of tension riser decreases with the increase of flow velocity.

**Figure 12:**
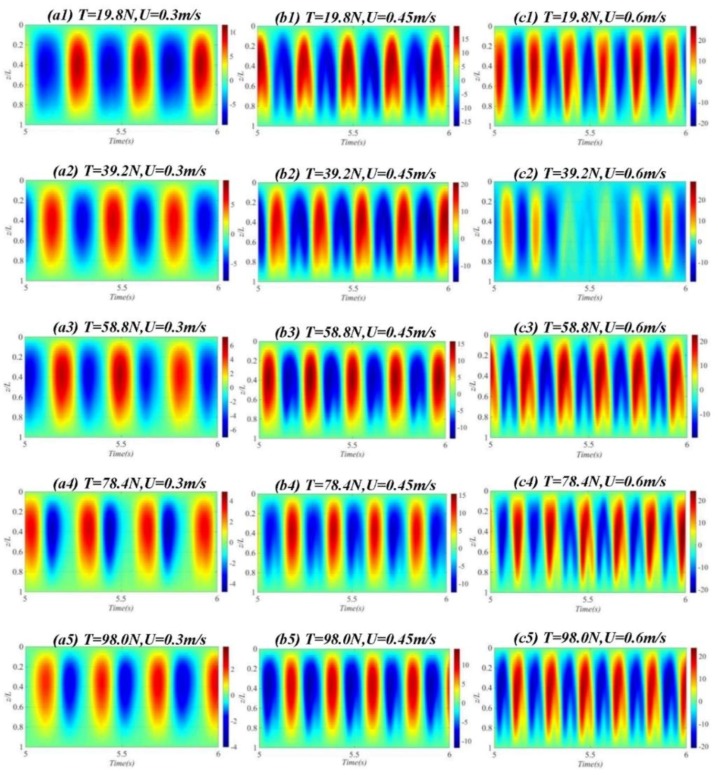
Time-varying graphs of riser dimensionless amplitudes at U = 0.3 m/s, U = 0.45 m/s, U = 0.6 m/s under different top tensions. (**a1**–**a5**) *T* = 19.8–98.0 N, *U* = 0.3m/s, dimensionless amplitude time-varying diagram of riser; (**b1**–**b5**) *T* = 19.8–98.0 N, *U* = 0.45 m/s, dimensionless amplitude time-varying diagram of riser; (**c1**–**c5**) *T* = 19.8–98.0 N, *U* = 0.6 m/s, dimensionless amplitude time-varying diagram of riser.

Standing wave effect describes a mode of vibration in which the vibration response varies temporally, whereas traveling wave effect describes a mode of vibration in which the vibration response varies both spatially and temporally. Bai et al. [27] observed that stable vibration amplitude exists in a standing wave region, with no obvious variation along the axis of the riser, whereas obvious displacement can be observed in the traveling wave region along the axis of the riser. In cross-flow direction, vibration is dominated by standing waves, whereas in in-line direction, standing waves occur on the boundary and in the still regions and these waves are passed from the high-velocity region toward the low-velocity region of stepped currents. Figure 12 shows the time-varying graphs of the riser’s dimensionless vibration amplitude under different top tensions. Only standing waves existed under the experimental cases. At *T* = 19.8–98.0 N, when 0.3 m/s < *U* < 0.6 m/s, as top tension increased, the riser’s vibration amplitude gradually decreased; as external flow velocity increased, the riser’s vibration changed from first-order mode to second-order mode [28,29] and the vibration intensity increased. The riser’s standing wave effects across the external flow velocities and top tensions were compared. Longitudinal analysis of these graphs reveals that top tension variation did not make much difference to the standing wave effect. Horizontal analysis discovered that external flow variation made a remarkable difference to the standing wave effect. The standing wave is in a transitional period of transformation to traveling wave. At the same velocity, the standing wave effect of the opposite riser is weak when the top tension increases.

### 4.3. Cross-Flow and in-Line Coupling

**Figure 13:**
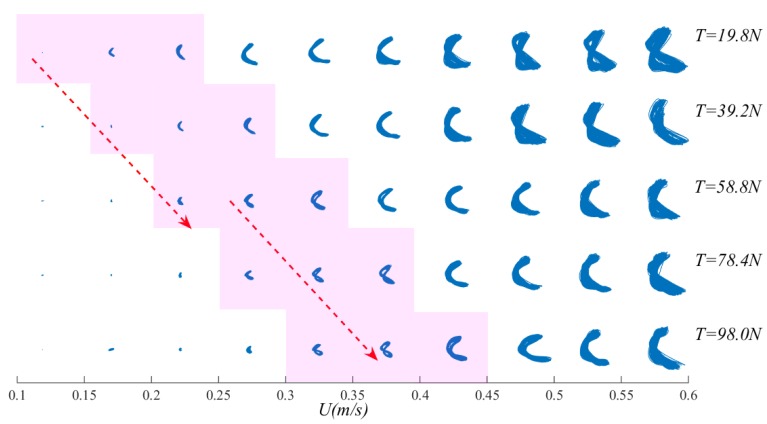
Riser vibration trajectories versus flow velocity under different top tensions.

Vandiver [30] observed that the drag force of the in-line causes the bending deformation of the riser when the vortex excitation vibration occurs except the cross-flow periodic vibration caused by the loss of the vortex. At the same time, the vortex-induced vibration will cause the structure to vibrate upward in-line, and the in-line vibration amplitude is generally smaller than the cross-flow vibration amplitude. Cross-flow vibration of the riser is highly correlated to in-line vibration. From these graphs, at T = 19.8–98.0 N, when the flow velocity was low, the riser’s vibration displacement trajectory was quite small. When U = 0.35 m/s, the riser’s vibration trajectory first approximated an “8”. As flow velocity increased, the “8” disappeared and turned into a “crescent”. At T = 19.8 N, as external flow velocity increased, the riser’s vibration displacement trajectory became a standard “8”, meaning the vibration amplitude increased. At T = 39.2–98.0 N, when U < 0.45 m/s, as external flow velocity increased, the riser’s vibration displacement trajectory turned from a “spot” into an “8”. After that, when U > 0.45 m/s, the riser’s vibration amplitude increased, the displacement trajectory became a “fried dough twist” and this “fried dough twist” grew even more obvious with increasing top tension. As the top tension increases step by step, it is found that the “8” shape of the riser vibration track lags behind. Where “lag” refers to the delay phenomenon of the “8” shape of the vibration track of the riser with the increase of the top tension under the comparison of the top tension of the adjacent two stages in Figure 13.

**Figure 14:**
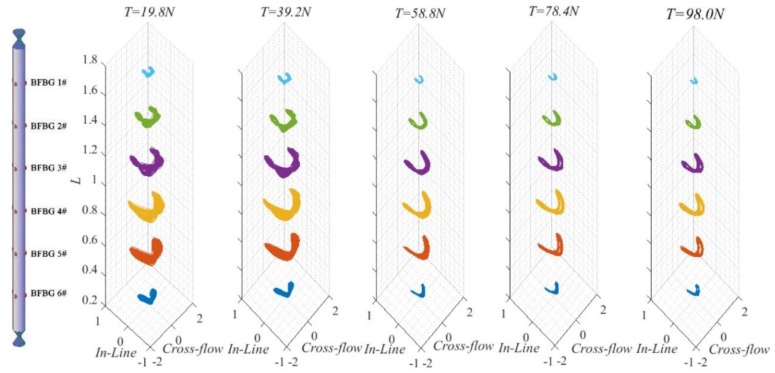
Three-dimensional riser axial motion trajectories at *U* = 0.45 m/s under different top tensions.

Figure 14 shows the motion trajectories at measuring points along the length of the riser when *U* = 0.45 m/s under different top tensions. As top tension increased, the displacement trajectory gradually reduced. Increasing top tension caused the riser’s vibration frequency to increase, thereby reducing its vibration displacement. At *T* = 19.8–58.8 N, along the axis of the riser, it is discovered that the riser’s displacement trajectory maximized somewhere near the center of the riser, since the riser’s vibration at this time was dominated by first-order mode. Taking the center of the riser as the dividing line, the closer a location is to the ends of the riser, the smaller the trajectory becomes; the closer a location is to the center of the riser, the more standard the “8” becomes. However, the part above the central point is not fully symmetrical to the part below the central point; the part of the riser in the flow field displayed a “fried dough twist” shape. The trajectories at the ends are different because the riser was not completely submerged in the uniform flow. Instead, the upper part is exposed to open air while the lower part is in a flow field. The different damping states and extra water masses between the above-water part and underwater part have accounted for the different vibration amplitudes and trajectories.

## 5. Conclusions

A vortex-induced vibration (VIV) experiment on a standing variable-tension riser was conducted in the combined wave–current water flume of the Engineering Hydrodynamics Laboratory, Ocean University of China to investigate the applicability and sensitivity of Bare Fiber Bragg Grating (BFBG) sensor technology for testing vibrations of deepsea risers and other slender marine structures. BFBG sensors were used to observe the dominant frequencies, dimensionless displacements, and in-line and cross-flow coupling of the riser VIV by varying the external flow velocity and top tension and using wavelet transform (WT) and modal decomposition. The following conclusions have been drawn:

(1) By changing the initial top tension of the five-level small difference, the BFBG sensing technology is used to measure the dynamic response of the riser strain, displacement, frequency and cross-flow and in-line coupling under the top tension. It is reflected that in the test of top tension deepsea riser VIV, BFBG sensing technology has good sensitivity and can clearly reflect the riser mode characteristics and vortex-induced vibration evolution process accompanying the flow velocity process. The Slalom Surround Installation approach can effectively prevent local breakage of the fiber optic string and competently ensure the operational stability of the bare fiber grating sensor in the experimental testing. Considering the diversity and complexity of the marine environment, the improvement of the flexural strength of the fiber and the development of new coating materials are crucial for the long-term stability of the BFBG sensor in the marine environment. 

(2) The in-line and cross-flow dominant frequencies of the riser gradually decreased across the same flow velocities with increasing top tension. Third- and higher-order vibration frequencies were very weak, whereas first- and second-order vibrations had a high involvement. As external flow velocity increased, the second-order frequency intensified remarkably, and the high-order harmonic components and “bimodal” pattern in the riser’s frequency response gradually disappeared with increasing top tension. In terms of displacement response, increasing top tension caused the RMS displacement of the riser to decrease and its amplitude to deteriorate. Since the riser has a tendency to change from the first-order mode to the second-order mode, at *U* > 0.55 m/s, *T* = 19.8 N, 39.2 N, the root mean square of the riser displacement suddenly drops, and the vibration intensity gradually enhanced. 

(3) Under the high flow velocity excitation, the increase of the top tension has a weak influence on the standing wave effect of the riser, and the standing wave gradually shows the transition trend to the traveling wave. With the riser center as the boundary, the closer to the end of the riser, the smaller the trajectory, and the more standard the “8” shape is. Due to the difference between the damping state of the flow field and the additional water quality, the vibration trajectories of the upper and lower parts of the riser center point are not strictly symmetrical, and the vibration trajectory of the lower riser has a “twist” shape. As the top tension increases, the riser vibration trajectory appears “lag”.

## Figures and Tables

**Table 1:** Detailed parameters of the riser.

Parameter	Unit	Value
Total length ***L***	m	2.0
Outer diameter ***D***	mm	18.0
Wall thickness ***δ***	mm	1.0
Mass per unit length ***m***	Kg	0.065
Sectional area ***S***	mm^2^	53.41
Submerged length ***Ls***	m	0.8 (40%)
Slenderness ratio (***λ*** =***L**/**D***)	-	111.11
Elasticity modulus ***E***	GPa	2.391
Bending rigidity ***EI***	N·m^2^	4.63
Material ***M***	-	PMMA

**Table 2:** Experimental cases.

Top Tension (N)	Serial Number (m/s)	Inherent Frequency (Hz)
19.8	0.1–0.6	3.408
39.2	0.1–0.6	4.354
58.8	0.1–0.6	5.139
78.4	0.1–0.6	5.801
98.0	0.1–0.6	6.403

## References

[1] Gedikli E.D., Dahl J.M. (2017). Mode excitation hysteresis of a flexible cylinder undergoing vortex-induced vibrations. J. Fluids Struct..

[2] Leclercq T., Delangre E. (2018). Vortex-induced vibrations of cylinders bent by the flow. J. Fluids Struct..

[3] Chaplin J.R., Bearman P.W., Huera-Huarte F.J., Pattenden R.J. (2005). Laboratory measurements of vortex-induced vibrations of a vertical tension riser in a stepped current. J. Fluids Struct..

[4] Lie H., Kaasen K.E. (2006). Modal analysis of measurements from a large-scale VIV model test of a riser in linearly sheared flow. J. Fluids Struct..

[5] Huera-Huarte F.J., Bearman P.W. (2009). Wake structures and vortex-induced vibrations of a long flexible cylinder—Part 1: Dynamic response. J. Fluids Struct..

[6] Huera-Huarte F.J., Bearman P.W. (2009). Wake structures and vortex-induced vibrations of a long flexible cylinder—Part 2: Drag coefficients and vortex modes. J. Fluids Struct..

[7] Lee L., Allen D. (2010). Vibration frequency and lock-in bandwidth of tensioned, flexible cylinders experiencing vortex shedding. J. Fluids Struct..

[8] Srinil N. (2011). Analysis and prediction of vortex-induced vibrations of variable- tension vertical risers in linearly sheared currents. J. Appl. Ocean Res..

[9] Gao G.H., Cui Y.J., Qiu X.Q., Shu Q. (2018). Analysis of natural vibration characteristics of deep-sea full-size tension riser. Pet. Mach..

[10] Li X.M., Zhang L., Niu J.J., Han Y.Q., Guo H.Y. (2016). Dynamic response analysis of deep-sea tension riser based on vector finite element method. Vib. Shock.

[11] Zhang J., Tang Y.G. (2014). Study on Mathieu instability of deepsea top tension riser. Ship Mech..

[12] Zhang W., Chen M.Z. (2019). An experimental study on the characteristic pattern of internal solitary waves in optical remote-sensing images. Int. J. Remote Sens..

[13] Hong l., Liu Z.W., Zhu Y., Zheng B., Liu F.X. (2018). Experimental study on an FBG strain sensor. Opt. Fiber Technol..

[14] Campanella C.E., Cuccovillo A.C., Clarissa Y.A., Passaro V.M.N. (2018). Fibre Bragg Grating Based Strain Sensors: Review of Technology and Applications. Sensors.

[15] Leal-Junior A.G., Frizera A., Daz C.R., Ribeiro M.R., Pontes M.J. (2018). FBG-embedded oblong diaphragms with extended dynamic range. IEEE Sens. Lett..

[16] Yuan Q., Wang Z.Y., Song L.P., Lu Z.Y., Hu D.N., Qin J.Q., Yang T.X. (2019). A Fast Linearly Wavelength Step-Swept Light Source Based on Recirculating Frequency Shifter and Its Application to FBG Sensor Interrogation. Sensors.

[17] Morey W.W., Meltz G., Glenn W.H. (1990). Fiber optic Bragg grating sensors. Proc. SPIE.

[18] Othonos A. (1997). Fiber bragg gratings. Rev. Sci. Instrum..

[19] Huang J., Zhou Z., Zhang L., Chen J., Ji C., Pham D.T. (2016). Strain modal analysis of small and light pipes using distributed fibre bragg grating sensors. Sensors.

[20] Peng J., Jia S., Jin Y., Xu S., Xu Z. (2019). Design and investigation of a sensitivity-enhanced fiber Bragg grating sensor for micro-strain measurement. Sens. Actuators A Phys..

[21] Song J., Lu L., Teng B., Park H., Tang G., Wu H. (2011). Laboratory tests of vortex-induced vibrations of a long flexible riser pipe subjected to uniform flow. Ocean Eng..

[22] Huera-Huarte F.J., Bangash Z.A., González L.M. (2014). Towing tank experiments on the vortex-induced vibrations of low mass ratio long flexible cylinders. Fluids Struct..

[23] Xu W., Cheng A., Ma Y., Gao X. (2018). Multi-mode flow-induced vibrations of two side-by-side slender flexible cylinders in a uniform flow. Mar. Struct..

[24] Blevins R.D. (1977). Flow Induced Vibration.

[25] Xu P., Hao W.S. (2016). Vibration Signal Processing and Data Analysis.

[26] Zhu H.J., Lin P.Z., Gao Y. (2019). Vortex-induced vibration and mode transition of a curved flexible free-hanging cylinder in exponential shear flows. J. Fluids Struct..

[27] Bai X., Le Z.B. (2018). Study on the traveling wave effect of vortex-induced vibration of a long flexible tube under the action of stepped flow. Ship Mech..

[28] Gedikli E.D., Chelidze D., Dahl J.M. (2018). Observed mode shape effects on the vortex-induced vibration of bending dominated flexible cylinders simply supported at both ends. J. Fluids Struct..

[29] Li P. (2012). Study on Vortex-Induced Vibration and Interference Experiment of Deep-Water Marine Conveyance Riser. Ph.D. Thesis.

[30] Vandiver J.K., Yong J.Y. (1987). The relationship between in-line and cross-flow vortex-induced vibration of cylinders. J. Fluids Struct..

